# Smoking and presence of human papillomavirus correlates with lymphocyte density in the stroma of penile squamous cell carcinoma

**DOI:** 10.3389/fonc.2025.1568764

**Published:** 2025-03-31

**Authors:** Chibamba Mumba, Victor Mapulanga, Nicholas K. Mwale, Owen Ngalamika

**Affiliations:** ^1^ Department of Pathology and Microbiology, University of Zambia School of Medicine, Lusaka, Zambia; ^2^ Urology Section, Department of Surgery, University of Zambia School of Medicine, Lusaka, Zambia; ^3^ Department of Physiological Sciences, University of Zambia School of Medicine, Lusaka, Zambia; ^4^ Dermatology and Venereology Division, University of Zambia School of Medicine, Lusaka, Zambia

**Keywords:** penile squamous cell carcinoma, tumor microenvironment, stroma, lymphocytes, HIV, human papillomavirus, smoking

## Abstract

**Background:**

Penile squamous cell carcinoma (PSCC) is the most common malignancy of the penis. Considering the increase in incidence of PSCC in many countries, there is a need for better and effective therapies for these patients. The tumor microenvironment may offer insights into a better understanding of the tumor, which may inform on predictive and prognostic targets. In this study, we investigated immune cell infiltration into the stroma of PSCC, and how it may be affected by multiple factors including smoking, HIV infection, and/or HPV infection.

**Methods:**

We carried out a prospective analytical cross-sectional study at the University Teaching Hospital in Lusaka, Zambia. Consenting patients with confirmed PSCC, attending the Urology Clinic and scheduled for partial or total penectomy were enrolled into the study. HIV testing by serology, HPV detection and genotyping on fresh tumors by real time PCR, hematoxylin and eosin (H&E) staining and immunohistochemistry staining for CD3^+^ and CD8^+^ cells on formalin-fixed paraffin-embedded tissue, and flow cytometry for immunophenotyping circulating immune cells were done.

**Results:**

We enrolled 33 participants into the study. The participants had a mean age of 56 years, the majority (84.8%) were HIV positive, high-risk HPV was detected in 63.6% of the tumors, and 57.6% were positive for both HIV and high-risk HPV. HPV-positive PSCC tumors had a significantly lower proportion of infiltrating lymphocytes in the stroma on H&E staining than HPV-negative tumors [18% vs 53%; p=0.025]. Smokers had a significantly lower number of infiltrating CD8^+^ cells in the stroma than non-smokers [68 vs 99; p=0.035]. No difference in the density of stromal lymphocytes between HPV/HIV co-infected and non-co-infected individuals was observed. There was a statistically significant positive correlation in number of CD3^+^ (⍴=0.38; p=0.027) and CD8^+^ (⍴=0.40; p=0.02) cells in the stroma and intra-tumor. Factors including histological stage, tumor grade, HPV status, and HIV status seem to influence the correlation in number of stroma and intra-tumoral immune cells.

**Conclusion:**

Smoking and presence of hrHPV is associated with a lower density of infiltrating lymphocytes in the stroma of PSCC. There is a positive correlation in the number of CD3^+^ and CD8^+^ cells between the stroma and intra-tumoral compartment of PSCC.

## 1 Introduction

Squamous cell carcinoma is the most common histological morphotype of cancer of the penis (1). Penile squamous cell carcinoma (PSCC) has a low incidence in developed countries, less than 1 per 100,000 males, while in other parts of the world including South America, Southeast Asia and Africa, have higher incidence rates ranging from 1-8 per 100,000 males (2, 3). However, several developed countries are also observing an increase in age-standardized incidence rates for PSCC (2).

High-risk Human Papilloma virus (hrHPV) is an important risk factor for penile cancer, and was detected in about a third of penile cancers from multiple regions of the world, including a few sub-Saharan Africa (SSA) countries (4). In geographic locations with high HPV prevalence, like some countries in SSA, penile cancer rates are higher and tend to affect younger individuals (5). Infection with HIV is another important risk factor for PSCC development and progression, with a relative risk of 3.7 to 5.8 among infected individuals, and a 4-fold increased risk of death among HIV-infected PSCC patients (6). The high prevalence of both HIV and HPV infections in SSA may explain the reason for a high incidence of PSCC, and especially affecting younger males, in this region (7).

Unfortunately, a majority of PSCC patients in SSA present with advanced stage disease, which is often associated with a poor prognosis (8). Surgical treatment and other treatment modalities such as chemotherapy may not be indicated or may be ineffective in management of advanced PSCC (9). Therefore, effective targeted therapies, especially immune therapy, may be more effective in treating such patients with advanced disease. The tumor immune microenvironment may offer insights into possible immunotherapeutic options for PSCC patients (10).

Although previous studies have reported on the infiltration of immune cells and expression of immune checkpoints in PSCC tumors, there is paucity of data on whether stromal tumor infiltrating lymphocytes (sTILs) correlate with treatment outcomes, clinical and histologic tumor characteristics, and/or intra-tumoral infiltration of immune cells in PSCC tumors. In luminal breast cancer, unfavorable histologic tumor characteristics, such as high histological grade and proliferative index, have been shown to associate with a higher percentage of sTILs (11, 12). However, in HER-2 positive and triple negative breast cancer, a high number of sTIL has been associated with better pathological complete response to neoadjuvant chemotherapy, and better invasive disease-free survival (13–15).

In this study, we evaluated the density of immune cells including all lymphocytes, CD3^+^, and CD8^+^ cells in the stroma of PSCC tumors. We investigated whether known risk factors including HIV infection, HPV infection, and smoking, and tumor characteristics including grade and stage associate with the density of sTILs. We also determined whether the number of sTILs correlates with the number of intra-tumoral immune cells.

## 2 Materials and methods

### 2.1 Study design and participants

This was a prospective analytical cross-sectional study, conducted at the Urology section of the department of surgery at the University Teaching Hospital in Lusaka, Zambia. Ethical approval was obtained from the University of Zambia Biomedical Research Ethics Committee (Ref. No.: 3233–2022) and the Zambia National Health Research Authority (Ref No.: NHRA0000010/31/10/2022). Participants were only recruited upon obtaining informed written consent between December 2022 and December 2023, and the study was performed in accordance with the declaration of Helsinki. Sociodemographic and clinical information were obtained from the patient using a questionnaire. Penile squamous cell carcinomas were collected after surgical treatment of primary tumor by either partial or total penectomy, processed in the pathology laboratory at UTH, stained with hematoxylin and eosin (H&E), and then histologically typed, graded and staged. Approximately 18mls of venous whole blood was also collected preoperatively.

### 2.2 HIV viral load and CD4 counts

A portion of the collected whole blood at baseline was subjected to HIV testing, HIV viral load determination, and CD4 counting. HIV-1 viral load was determined using the Aptima HIV-1 Quant Dx Assay kit (Hologic) on the Hologic Panther, according to the manufacturer’s instructions. HIV viral load levels below the lower limit of detection (<30 copies/ml) were recorded as zero for analytical purposes. The BD FACSCalibur instrument (BD Biosciences) was used to determine the CD4 counts using the BD TriTest kit (BD Biosciences) on 50µL whole blood, according to manufacturer’s instructions. These are part of routine investigations for HIV-infected individuals, and are done at a dedicated laboratory at the institution.

### 2.3 Flow cytometry

A portion of the peripheral blood was also subjected to peripheral blood mononuclear cell (PBMC) isolation by density gradient centrifugation. The PBMCs were then subjected to CD4 and CD8 T cell sorting on an automated cell sorter (AutoMACS, Miltenyi Biotec). The sorted cells were then stained with monoclonal antibodies against the following surface markers: CD3 APC-Vio770, CD45RO APC, CCR7 PE-Vio770, PD-1 BB515, and CD69 PERCP. Upon incubation in the dark for 20 minutes at room temperature, the cells were washed, resuspended in phosphate-buffered saline, and evaluated by flow cytometry on a BD FACSVerse instrument. Fluorescence-minus-one controls were used to define the gates, and analysis was done using Flow jo version 10. The gating strategies are shown in **Supplementary Figure 1**. Briefly, gating was done on sorted CD4 and CD8 cells. Then CD3^+^ cells were gated on, followed by phenotypic characterization of T cell subsets based on their expression of CD45RO and CCR7. T cell subsets included naïve (CD45RO^-^CCR7^+^), central memory (CD45RO^+^CCR7^+^), effector memory (CD45RO^+^CCR7^-^) and Terminally-differentiated effector cells (TEMRA) (CD45RO^-^CCR7^-^). Gating was also done on CD3^+^ cells expressing PD-1 and CD69.

### 2.4 HPV detection and genotyping

HPV detection and genotyping was done on fresh PSCC tumors. DNA was first extracted using the QIAampRDNA Mini Kit. We then detected and genotyped HPV using the AnyplexTM II HPV28 detection kit by qPCR on the CFX96TM Real-time PCR detection system (BIO-RAD). The kit allows for the detection of 28 different HPVs that include 19 high-risk (16, 18, 26, 31, 33, 35, 39, 45, 51, 52, 53, 56, 58, 59, 66, 68, 69, 73, and 82) and 9 low-risk (6, 11, 40, 42, 43, 44, 54, 61, and 70) HPV genotypes.

### 2.5 Immunohistochemistry and cell enumeration

Immunohistochemical (IHC) staining of formalin-fixed paraffin-embedded penile tumors was performed using a semi-automated slide preparation system. We stained the tumor using antibodies against CD3 and CD8. Mononuclear cells with morphological characteristics of lymphocytes were enumerated. Briefly, four (4) micrometer sections were mounted on adhesive slides (Leica Biosystems). During each staining, we included palatine tonsil or lymph-node as positive controls, while the negative control was tissue incubated without the primary antibody. After baking the samples and controls for 1-2 hours at 60°C, the semi-automated PT Link (Agilent) was used for antigen retrieval according to manufacturer’s guidelines. The primary antibody was then added to the samples, after some washing and blocking steps, and left for an hour. The samples were then washed in wash buffer and then incubated with a post primary linker (Novolink polymer detection; Leica Biosystems) for 30 minutes. Peroxidase activity was developed using Diaminobenzidine working solution. Hematoxylin was then finally applied as a background stain.

The IHC stained slides and corresponding hematoxylin and eosin (H&E) stained slides were assessed by an experienced reviewer, and the density of stromal tumor infiltrating immune cells (sTILs) was assessed microscopically at high power magnification (x400) in five different fields that were representative of the tumor stroma, which included the invasive front and intra tumoral stroma. The count was expressed as an average absolute count (for CD3 and CD8 IHC) and average percentage of stroma infiltrated by mononuclear cells with morphological features of lymphocytes (for H&E). Counting was also done in the intra-tumoral compartment and expressed as count in a high-power field.

### 2.6 Data analysis

Baseline characteristics were analyzed using summary statistics. Since the absolute cell counts and percentages were non-normally-distributed continuous variables, comparisons of these variables between dichotomous groups (e.g. by high-risk HPV (hrHPV), HIV, or smoking status) was done using the Mann-Whitney test. Correlation between the number of sTILs and number of intra-tumoral immune cells was done using the Spearman’s rank correlation (Rho (⍴)). Multivariate analysis was not done due to the small sample size. P values less than 0.05 were considered statistically significant.

## 3 Results

### 3.1 Baseline characteristics

Thirty-three (33) participants with PSCC were enrolled into the study. The participants had a median age of 56 years, 24% were below 50 years, 36% were below 60 years, and the youngest was 29 years old. The majority (84.8%) of the participants were HIV positive, all HIV^+^ individuals were on anti-retroviral therapy, and about half of the participants (51.5%) were smokers. High-risk HPV was detected in 63.6% of all the tumors, and all HPV-positive tumors had high-risk HPV. The rest of the baseline characteristics are shown in **Table 1**.

**Table 1:** Baseline characteristics of study participants.

Age (Years)	56 [50-62]
Smoking	17/33 (51.5%)
HIV Positive	28/33 (84.8%)
Advanced Histological Stage	8/33 (24.2%)
High-Grade Lesion	6/14 (42.9%)
On ART (for HIV^+^)	28/28 (100%)
CD4 Count (cells/µL)	467 [310-656]
HIV Viral Load (Copies/mL)	0 [0-0]
High-Risk HPV Detected	21/33 (63.6%)

ART, Antiretroviral Therapy; Normal reference range for CD4 count was 410-1590cells/µL.

### 3.2 Factors associated with stromal T-cell density in PSCC

**Figure 1:**
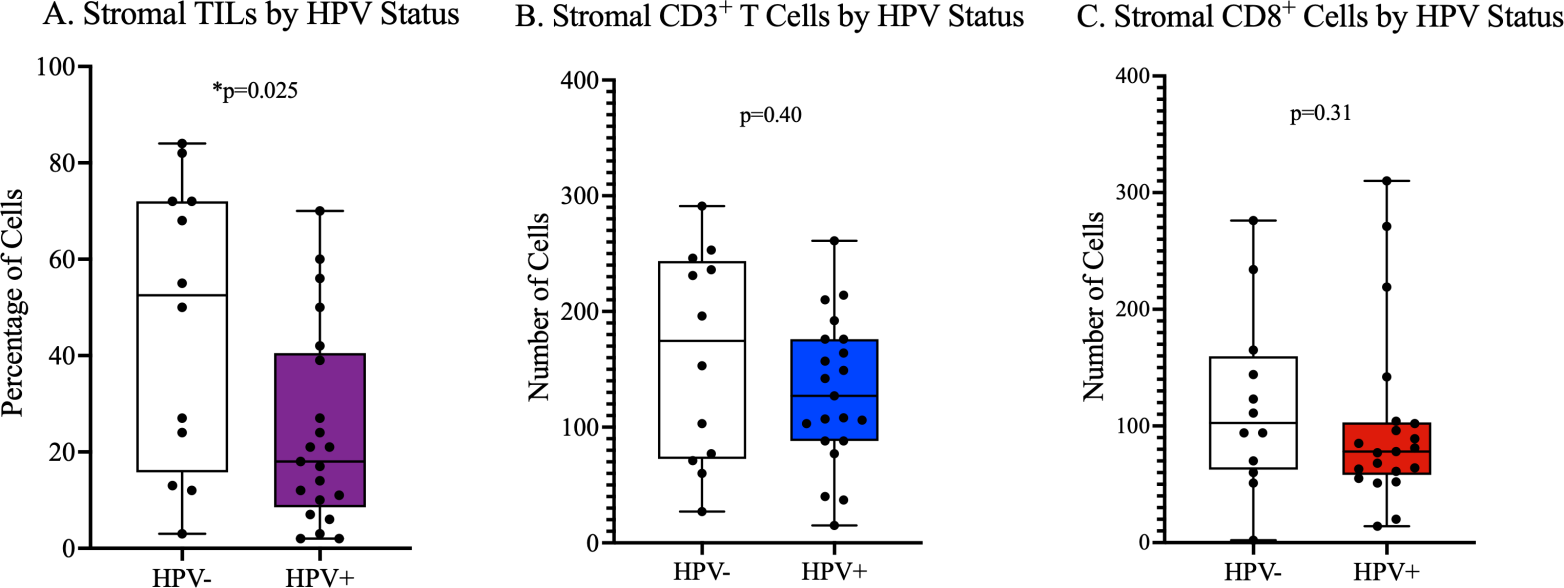
Stromal tumor infiltrating lymphocyte density by HPV status. **(A)** Statistically significantly higher density of sTILs in HPV^-^ tumors than HPV^+^ by hematoxylin and eosin-stained tumor evaluation. **(B, C)** Higher but statistically insignificant number of CD3^+^ and CD8^+^ cells in HPV negative tumors compared to HPV positive tumors, respectively.

**Figure 2:**
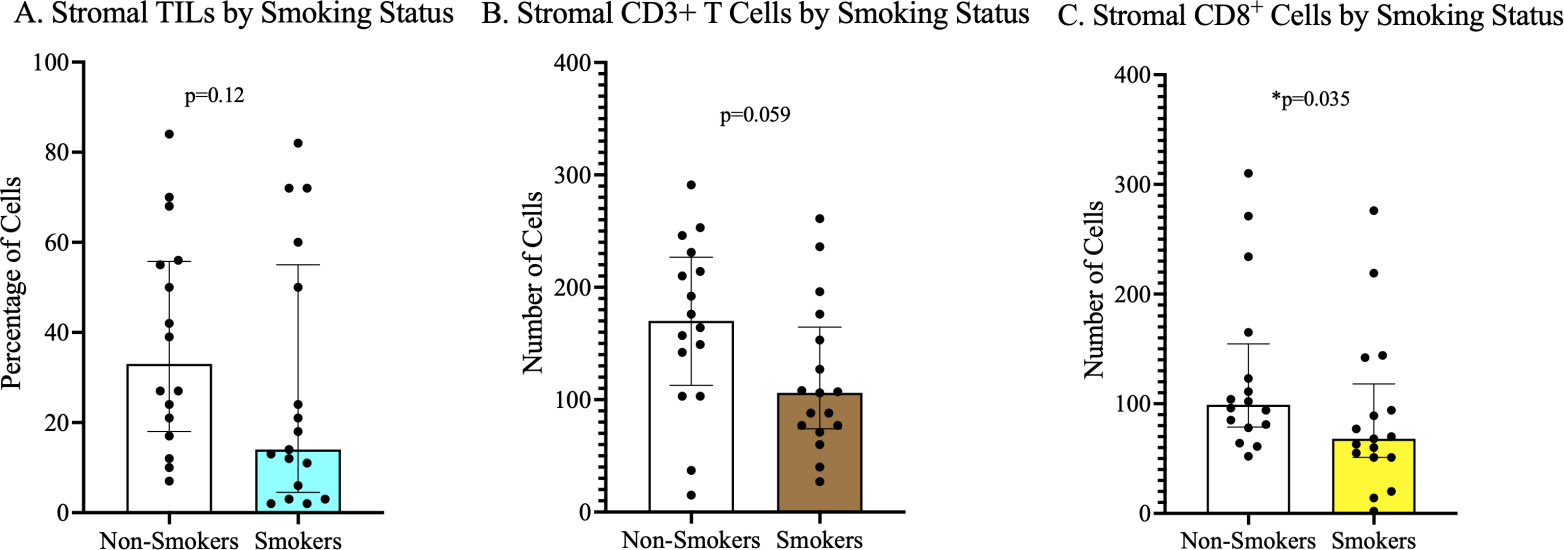
Stromal tumor infiltrating lymphocyte density by smoking status. **(A)** Higher but statistically insignificant stromal lymphocytes on H&E staining in non-smokers compared to smokers. **(B)** Higher but statistically insignificant stromal CD3^+^ cells in non-smokers versus smokers. **(C)** There was a statistically significantly higher number of CD8^+^ cells in PSCC tumors of non-smokers than in smokers.

We observed that the stroma of HPV-negative tumors had a statistically significant higher proportion of lymphocytes on hematoxylin and eosin-stained tumors than hrHPV-positive tumors (**Figure 1** and **Supplementary Figure 2**). We also observed on IHC staining that the stroma of PSCC patients with a history of smoking had a significantly lower number of CD8^+^ cells than non-smokers (**Figure 2**). No significant difference in number and proportion of immune cells was observed in PSCC stroma by HIV status, HIV/HPV co-infection, histological stage, and histological grade (**Supplementary Table 1**). Other parameters including age, size of the primary tumor, and duration of the lesion did not show any correlation with the number of stromal immune cells.

### 3.3 Correlation between stromal and intra-tumoral lymphocyte density

A higher absolute number of CD3^+^ and CD8^+^ cells in the tumor stroma positively correlated with a higher infiltrate in the intra-tumoral compartment, and the correlation was statistically significant (**Table 2** and **Supplementary Figure 3**). We also assessed parameters that could affect the correlation between the number of stromal lymphocytes and intra-tumoral lymphocytes (**Table 2**). We observed that early-stage tumors had a statistically significant positive correlation in number of CD3^+^ cells between the stroma and intra-tumoral compartment. We also observed that HPV^+^ tumors had a statistically significant positive correlation in the number of both CD3^+^ and CD8^+^ cells between the stroma and intra-tumoral compartment. High grade tumors showed a statistically significant positive correlation in proportion of lymphocytes between the stroma on H&E staining and number of CD3^+^ cells in the intra-tumoral compartment. HIV^+^ individuals had a statistically significant positive correlation in number of both CD3^+^ and CD8^+^ cells between the stroma and intra-tumoral compartment. On the other hand, HIV- individuals had a statistically significant negative correlation in number of CD3^+^ cells between the stroma and intra-tumoral compartment.

**Table 2:** Correlation between number of cells in stroma and intra-tumoral compartment.

	sTILs (%) and tCD3 Count	sCD3^+^ Count and tCD3 Count	sCD8^+^ Count and tCD8 Count	sCD8^+^ (%) and tCD8 Count
All	⍴=0.30; p=0.10	⍴=0.38; p=0.027	⍴=0.40; p=0.02	⍴=0.42; p=0.016
Early Stage	⍴=0.42; p=0.039	⍴=0.43; p=0.033	⍴=0.34; p=0.10	⍴=0.34; p=0.09
Advanced Stage	⍴=0.0001; p=1.0	⍴=0.42; p=0.30	⍴=0.62; p=0.10	⍴=0.67; p=0.07
Low Grade	⍴= -0.05; p=0.91	⍴=0.40; p=0.32	⍴=0.62; p=0.10	⍴=0.64; p=0.09
High Grade	⍴=0.87; p=0.025	⍴=0.72; p=0.10	⍴=0.26; p=0.62	⍴=0.31; p=0.54
HPV^-^	⍴=0.29; p=0.35	⍴=0.39; p=0.21	⍴=0.37; p=0.23	⍴=0.30; p=0.34
HPV^+^	⍴=0.12; p=0.61	⍴=0.43; p=0.05	⍴=0.33; p=0.14	⍴=0.45; p=0.04
HIV^-^	⍴=0.10; p=0.87	⍴= -0.90; p=0.037	⍴= -0.10; p=0.87	⍴=0.46; p=0.43
HIV^+^	⍴=0.42; p=0.027	⍴=0.48; p=0.01	⍴=0.49; p=0.01	⍴=0.47; p=0.01

TIL, Tumor infiltrating lymphocyte; ⍴=Rho/correlation coefficient; s: all lower case ‘s’ indicate “stroma”; t: all lower case ‘t’ indicate “intra-tumoral compartment”. Values in bold indicate significant p values.

A correlation between absolute counts and proportions for CD8^+^ T cells (**Table 2**) was also done for several reasons. Firstly, the number of cells in the intra-tumoral compartment is much lower than that in the stroma, hence it was not possible to obtain realistic proportions of infiltrating lymphocytes in the intra-tumoral compartment. Secondly, since the stroma has high number and diverse populations of immune cells, both the absolute and relative counts may be useful in assessing the correlation with the absolute number of immune cells in the intra-tumoral compartment. Thirdly, an expansion of a particular immune cell type (e.g. CD8^+^ T cells) relative to other cells, could be more informative with regards to the number of that particular cell population migrating into the tumor.

### 3.4 Characteristics of circulating T cells in smokers compared to non-smokers

**Figure 3:**
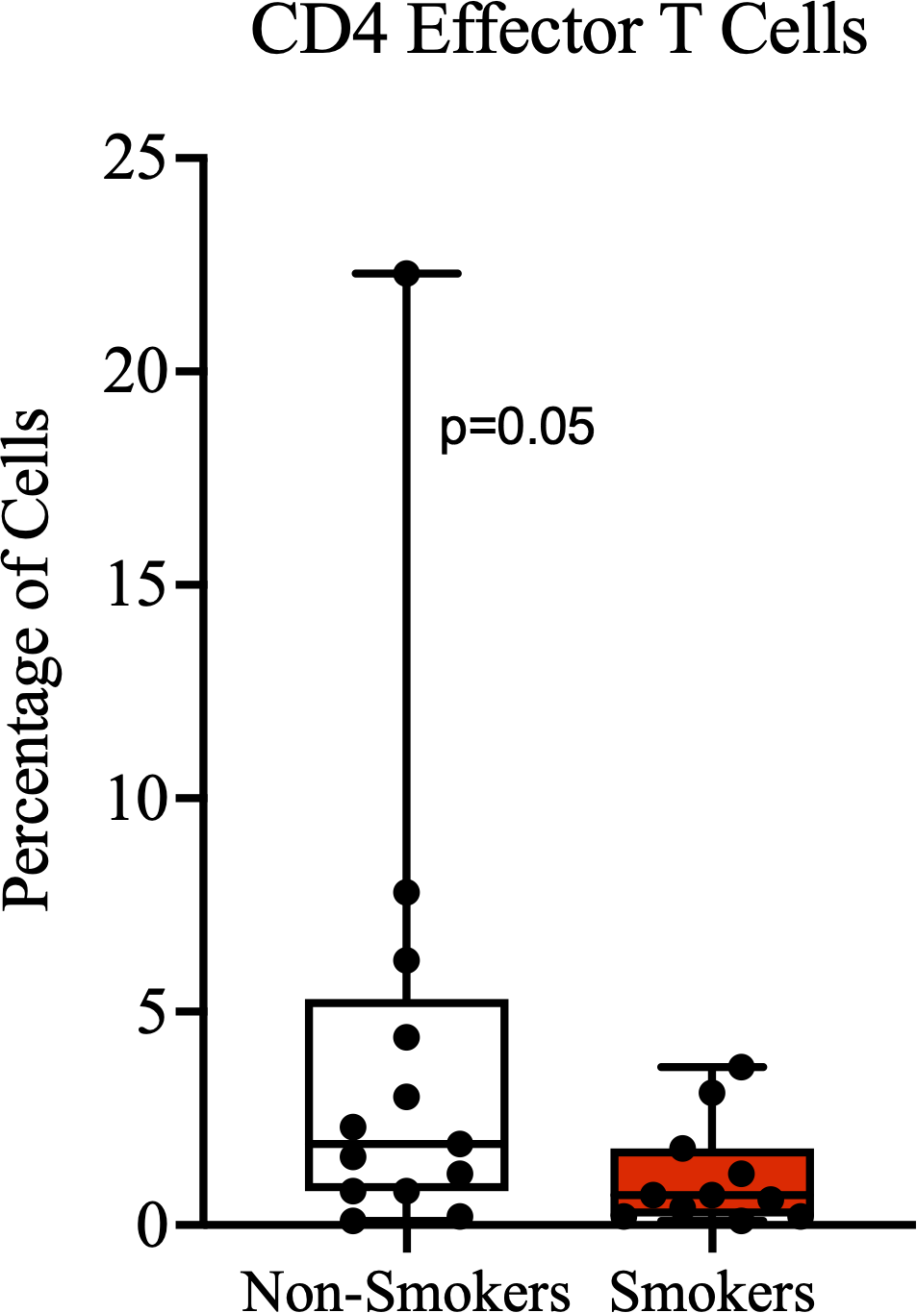
Effector CD4 T cells. A higher proportion of terminally-differentiated effector CD4^+^ T cells among non-smokers compared to smokers, with borderline significance.

Since we observed a significantly lower number of CD8^+^ cells in the stroma of individuals with a history of smoking compared to non-smokers, we further compared the characteristics of T cells in the blood circulation by smoking status. Age, HIV status, CD4 counts, proportion of CD8 T cell subsets, including proportion of cells expressing the immune checkpoint PD-1 were not significantly different between the groups (**Supplementary Table 2**). The proportion of CD4^+^ T cells expressing the homing and activation marker CD69 was higher among the non-smokers than the smokers, but this difference was not statistically significant (**Supplementary Table 2**). In addition, non-smokers had a borderline significantly higher proportion of circulating effector CD4^+^ T cells than non-smokers (**Figure 3**). Also, the median proportion of CD4 effector memory T cells was higher among non-smokers than smokers, although this difference was also not statistically significant.

## 4 Discussion

In this study, we have investigated the factors associated with the number and relative proportion of T-cells in stroma of PSCC. We have also investigated factors influencing the correlation between the number of T-cells in the stroma and the intra-tumoral compartment of PSCC. As expected, we had a high proportion (almost 25%) of individuals below the age of 50, and the median age was 56 years. This is a younger age at presentation compared to most developed countries where PSCC often occurs in elderly males above 60 years (16, 17).

In a previous study by Hladek L. et al., a report of significantly higher density of CD3^+^ immune cells in low-stage PSCC tumors when compared to advanced-stage tumors was made (18). In this study, we observed that tumors with a low histological stage had a higher number of CD3^+^ cells than those with an advanced stage. However, this observation was not statistically significant, possibly due to the small number of examined tumors. A high number of CD3^+^ immune cell infiltration has been shown to correlate with better survival in several cancers (19). On the other hand, there are subpopulations of T cells, such as CD3^+^/CD4^+^ regulatory T cells, that are known to promote tumor progression (20). We did not look at this and other T cell subsets in our study, and therefore we cannot make absolute conclusions on whether the actual differences between early and advanced tumors are in the number of subpopulations of T cells as opposed to absolute number of CD3^+^ T cells.

In this study, hrHPV-positive PSCC tumors had significantly lower numbers of lymphocytes in the stroma, on H&E staining, compared to hrHPV-negative tumors. Also, the numbers of CD3^+^ and CD8^+^ T cells were higher in hrHPV-negative than hrHPV-positive tumors, although this difference was not statistically significant. These findings may be supported by previous studies on immune evasion mechanisms of HPV, including observations that HPV oncoproteins E6 and E7 inhibit expression and signaling of interferons (IFN), which promote anti-viral immunity and apoptosis of cancer cells (21, 22). Also, hrHPV oncoproteins have been shown to downregulate pro-inflammatory cytokines such as IL-1β and chemokines such as CCL2 (23–25), which may lead to low infiltration of inflammatory cells in HPV-associated tumors. Some previous studies have reported findings similar to ours, of lower numbers of infiltrating T cells in the tumor environment of HPV-positive PSCC tumors than HPV-negative tumors (26), while others have reported higher numbers in HPV-positive tumors than HPV-negative tumors (27). For instance, several studies in head and neck cancers have reported higher TIL levels in HPV-positive tumors than HPV-negative tumors (28–30). Therefore, our findings require further validation with a larger sample size. Moreover, other co-existing factors including HIV infection, which showed higher presence of infiltrating lymphocytes in our study population, could influence the number of infiltrating immune cells in co-infected individuals. A plausible explanation for a significant difference in stromal TILs on H&E staining and not immunohistochemistry for CD3 and CD8 is that on H&E staining multiple cell types including T cells (multiple subsets), B cells, and natural killer cells are captured together. There could be differences in composition of the lymphocyte-appearing cells, other than CD8 cells alone, that could explain the differences by HPV status. Other studies have similarly observed that H&E staining may be more useful in assessing the density of stromal TILs and predicting prognosis for various cancers (31–34).

PSCC patients who had no history of smoking had significantly higher number of CD8^+^ cells in their tumor stroma compared to smokers. In a previous study on esophageal squamous cell carcinoma, it was also found and reported that tumor stroma of smokers had significantly lower number of activated cytotoxic T lymphocytes compared to non-smokers (35). Smokers are known to have a chronically activated inflammatory state way before the onset of conditions such as cancer (36). This early state of chronic immune activation is thought to ultimately result in exhaustion and depletion of immune cells (37, 38), which may affect anti-tumor immunity when a cancer develops. Other reported effects of smoking on T cells, that may impair anti-tumor immunity and surveillance, include the suppression of Granzyme B expression in CD8^+^ T cells, reduced activation of cytotoxic T lymphocytes, and promotion of senescence and exhaustion (39). The known effects of smoking on immune surveillance and our findings could be further supported by our other observation that non-smokers had a borderline significantly higher proportion of CD4 effector cells than smokers. Although we did not explore the proportion of cells expressing CD103, an important homing marker, it is possible that non-smokers may have more effector T cells destined for migration to the PSCC tumor environment.

The interaction and correlation between immune cells in the stroma and tumor space is important for anti-tumor immunity. A strong positive correlation between stromal and intra-tumoral lymphocytes has previously been associated with pathologic complete response after chemotherapy in breast cancer (40). In penile cancer, a study by Chu et al. has previously shown that number of immune cells, such as CD8-positive cells, in the stroma are positively correlated with number of the same cells in the intra-tumoral space (41). These observations are similar to ours where we found that the number of CD3^+^ and CD8^+^ cells in the stroma are positively correlated with those in the intra-tumoral compartment. However, this correlation seems to be affected by other factors. For instance, a significant correlation was observed in early stage and not advanced stage tumors, which could imply that as tumors advance, they acquire some characteristics that may hinder infiltration by immune cells. Also, high grade lesions showed a positive correlation between proportion of stromal TILs on H&E staining and number of intra-tumoral CD3^+^ cells, which may be a result on higher tumor infiltration by immune cells due to high mutational burden in high grade tumors (42). HPV^+^ tumors and an HIV^+^ status showed a significant positive correlation in number of immune cells in stroma and intra-tumor. On the other hand, tumors from HIV^-^ patients had a significant negative correlation between CD3^+^ cells in the stromal and intra-tumoral compartment. Hence, factors such as HPV and/or HIV status may influence the correlation between number of stromal and intra-tumoral immune cells. Therefore, HPV and/or HIV infection status, and lymphocyte density in the stroma of PSCC, may serve as predictive markers on patients likely to respond to treatments such as immune therapy.

This study has a number of limitations. The sample size may not have been adequate to address all the investigated immune parameters. Also, since we only stained for CD3^+^ and CD8^+^ T cells, evaluation on numbers of CD4^+^ T cells and/or other immune cells such as professional antigen presenting cells may have given a more complete picture on the immune cell population in the stroma of PSCC tumors. In addition, information on extent and duration of smoking was not collected. Therefore, we could not perform the correlation between chronic inflammation associated with smoking and duration of smoking. Another limitation was inability to assess regulatory T cells, as they are anti-inflammatory and could explain some of the observed tumor characteristics. Furthermore, assessing how stromal infiltration correlates with prognosis would have been more informative, despite the fact that all our study participants underwent surgical treatment with or without chemotherapy and/or radiotherapy, and none of the participants received immune therapy.

## 5 Conclusions

HrHPV-negative PSCC tumors have a higher number of stromal tumor-infiltrating lymphocytes, while smoking is associated with a lower number of stromal infiltrating lymphocytes. Factors such as early histological stage, high tumor grade, HIV-positivity, and HPV-positivity display a positive correlation between number of CD3^+^ and CD8^+^ cells in the PSCC stroma and intra-tumoral compartment.

## Data Availability

The raw data supporting the conclusions of this article will be made available by the authors, without undue reservation.
