# Supplementary material for: Smoking and presence of human papillomavirus correlates with lymphocyte density in the stroma of penile squamous cell carcinoma
Source: Front Oncol. 2025 Mar 31;15:1568764. doi: 10.3389/fonc.2025.1568764 (PMC11994432; doi:10.3389/fonc.2025.1568764)
Supplement: Supplementary file 1 [file DataSheet1.docx]

| **Supplementary Table 1:** Factors affecting number of lymphocytes in the stroma of PSCC tumors | | | | | |
| --- | --- | --- | --- | --- | --- |
|  | **sTILs Percent** | **sCD3^+^**  **Count** | **sCD3^+^**  **(%)** | **sCD8^+^**  **Count** | **sCD8^+^**  **(%)** |
| **Early Stage** | 27 [12-60] | 153 [88-214] | 31 [17-44] | 89 [55-123] | 23 [14-40] |
| **Advanced Stage** | 16 [10-24] | 117 [74-159] | 32 [18-53] | 81 [66-157] | 22 [15-42] |
| *P Value* | 0.12 | 0.22 | 0.76 | 0.77 | 0.94 |
| **Low Grade** | 42 [19-70] | 159 [49-212] | 38 [23-44] | 86 [66-113] | 27 [10-43] |
| **High Grade** | 24[12-50] | 132 [103-236] | 25 [15-60] | 108 [94-144] | 27 [10-55] |
| *P Value* | 0.47 | 0.85 | 0.88 | 0.30 | 0.95 |
| **HPV-** | 53 [19-72] | 175 [74-241] | 38 [20-44] | 103 [65-155] | 30 [15-55] |
| **HPV+** | 18 [10-39] | 127 [88-176] | 27 [17-42] | 78 [61-102] | 22 [14-30] |
| *P Value* | **0.025** | 0.40 | 0.31 | 0.31 | 0.40 |
| **HIV-** | 21 [13-72] | 88 [77-142] | 32 [17-38] | 70 [60-85] | 15 [14-15] |
| **HIV+** | 24 [12-53] | 153 [96-212] | 31 [17-45] | 92 [62-143] | 24 [16-48] |
| *P Value* | 0.80 | 0.20 | 0.86 | 0.21 | 0.15 |
| **No co-infection** | 38.5 [13-72] | 147.5 [77-236] | 38 [17-44] | 94 [60-144] | 22.5 [14-54] |
| **HIV/HPV co-infection** | 18 [10-42] | 127 [88-176] | 27 [17-46] | 78 [61-104] | 23 [14-32] |
| *P Value* | 0.12 | 0.58 | 0.50 | 0.66 | 0.94 |
| **Non-Smoker** | 33 [19-56] | 170 [123-223] | 35 [25-47] | 99 [80-144] | 28 [20-55] |
| **Smoker** | 14 [6-50] | 106 [77-153] | 19 [16-42] | 68 [51-94] | 17 [12-28] |
| *P Value* | 0.12 | 0.059 | 0.18 | **0.035** | **0.031** |
| TIL: Tumor infiltrating lymphocyte; s: all lower case s on T cell markers indicate “stroma”. Values in bold indicate significant p values. | | | | | |

Supplementary Material

# Supplementary Tables

| **Supplementary Table 2.** Circulating T-Cell Subsets in by Smoking Status | | | |
| --- | --- | --- | --- |
|  | **Non-Smokers** | **Smokers** | **p Value** |
| Age | 54.5[50.5-62.5] | 57[47-61] | 0.95 |
| HIV Positive | 93.8% | 76.5% | 0.17 |
| CD4 Count | 451 [245-651] | 619.5[484.5-729.5] | 0.14 |
| C Reactive Protein | 5[2.5-28.5] | 4.9[2.5-21.3] | 0.98 |
| HPV+ PSCC | 68.8% | 58.8% | 0.55 |
| Sorted CD4 cells | 12134[5085-13832] | 13101[5873-14488] |  |
| CD3^+^ cells among sorted CD4 cells | 7637[3269-13099] | 11439[4760-14198] |  |
| Naïve CD4 T Cells | 37.4%[10.5-49.2] | 44.7%[28.6-54.1] | 0.30 |
| CD4^+^ CM T Cells | 36.3%[25.9-62.6] | 45.7%[25.4-51] | 0.58 |
| CD4^+^ EM T Cells | 13.2%[9.8-23.8] | 8.6%[3-18.9] | 0.08 |
| CD4^+^ Effector T Cells | 1.9%[1.3-5.4] | 0.7%[0.7-2.5] | 0.05 |
| PD1^+^ CD4 T Cells | 38.4%[18.4-27.4] | 30.2%[15.6-21] | 0.23 |
| CD69^+^ CD4 T Cells | 6.1%[51.5-70.8] | 3%[1.3-61.7] | 0.12 |
| Sorted CD8 cells | 12835[9090-20325] | 12631[10390-17817] |  |
| CD3^+^ cells among sorted CD8 cells | 10710[7926-19777] | 11090[10088-15893] |  |
| Naïve CD8 T Cells | 20.3%[5.8-21.7] | 23.4%[12.4-32.2] | 0.29 |
| CD8^+^ CM T Cells | 14.4%[7.2-48.9] | 12.4%[9.8-28.9] | 0.37 |
| CD8^+^ EM T Cells | 34.9%[23.6-42.5] | 27.4%[18.1-48.3] | 0.62 |
| CD8^+^ Effector T Cells | 18.8%[4.9-44.5] | 17.5%[12.2-25.3] | 0.50 |
| CD69^+^ CD8 T Cells | 8.8%[61.1-76.1] | 8.5%[4.1-82] | 0.77 |
| PD1^+^ CD8 T Cells | 16.2%[7.9-25.2] | 17.6%[11.5-19.9] | 0.90 |
| CD4 count represents counts on whole blood, performed as standard of care for HIV patients (section 2.2 of manuscript). The proportions of the T cell phenotypes are based on absolute numbers of CD3^+^ cells among sorted CD4 and CD8 cells from PBMCs. CM=Central Memory; EM=Effector Memory. | | | |

# Supplementary Figures

**
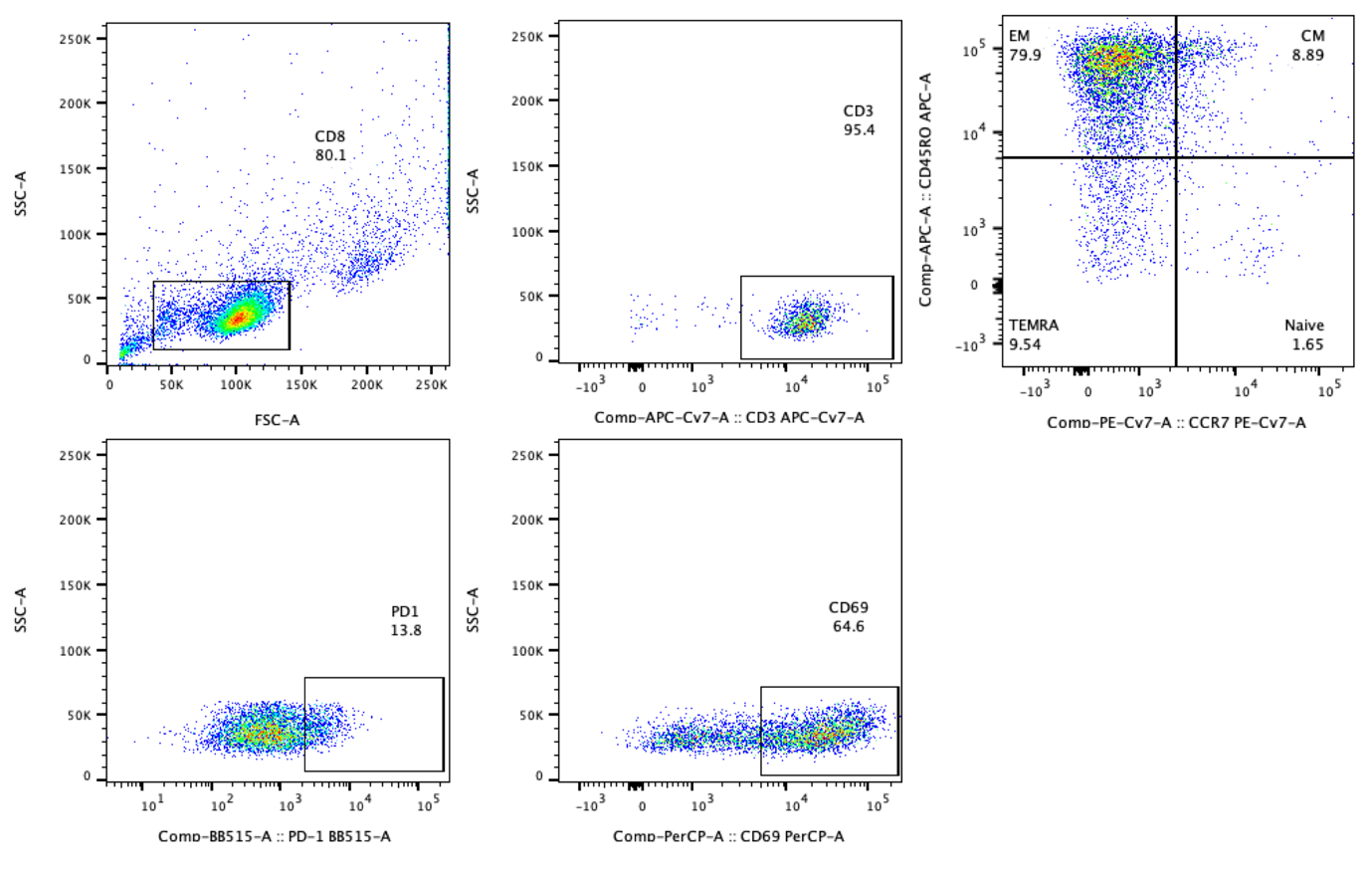
**

**Supplementary Figure 1**. Gating strategies for flow cytometry. From top right to top left then bottom left to bottom right, A population of sorted CD4 or CD8 cells were selected by forward and side scatter. Then gating was done on CD3^+^ cells, then T cell subsets by expression of CD45RO and CCR7. We then identified PD1^+^ and CD69^+^ cells among the CD3^+^ cells. Fluorescent minus-one controls were used to determine the boundaries. EM=Effector memory (CD3^+^CD45RO^+^CCR7^-^); CM=Central memory (CD3^+^CD45RO^+^CCR7^+^); TEMRA=Terminally differentiated effector cells (CD3^+^CD45RO^-^CCR7^-^); Naïve=CD3^+^CD45RO^-^CCR7^+^.

**
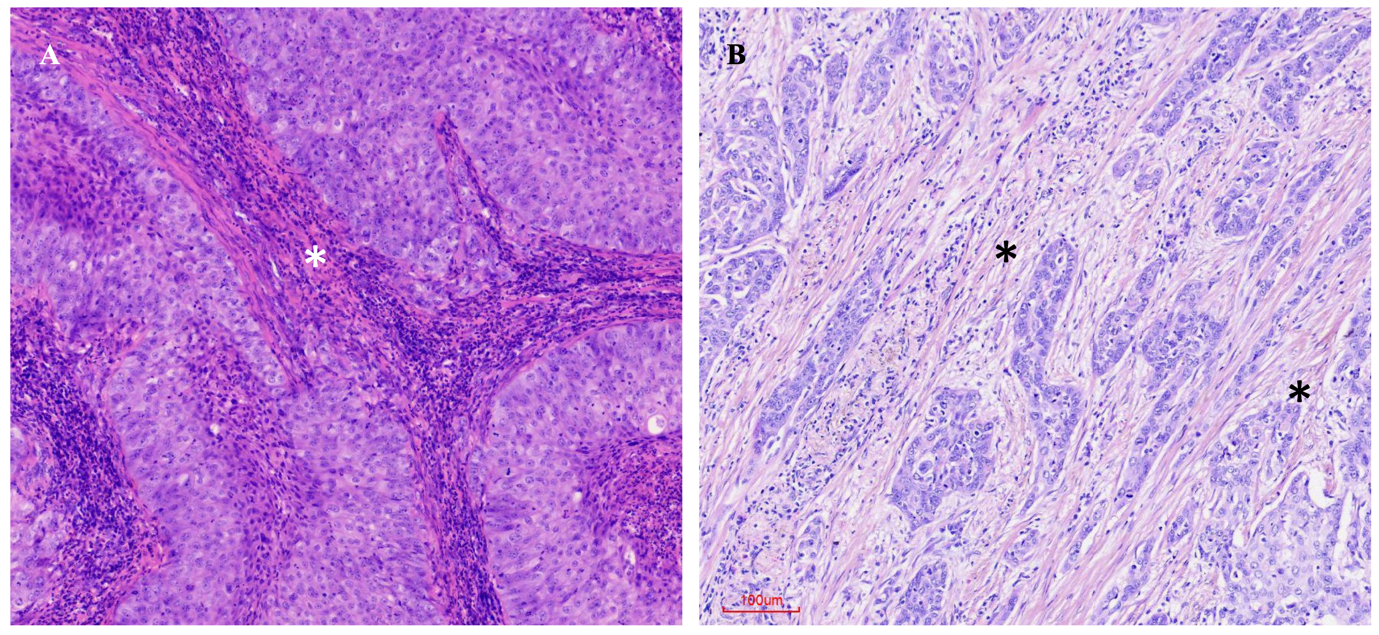
Supplementary Figure 2**. **A**) HPV^-^ H&E-stained PSCC (x10 magnification). White asterisk indicates a proportionately high infiltrate of mononuclear cells in the tumor stroma. **B**) HPV^+^ H&E-stained PSCC (x10 magnification). Black asterisks indicate a proportionately low infiltrate of mononuclear cells.


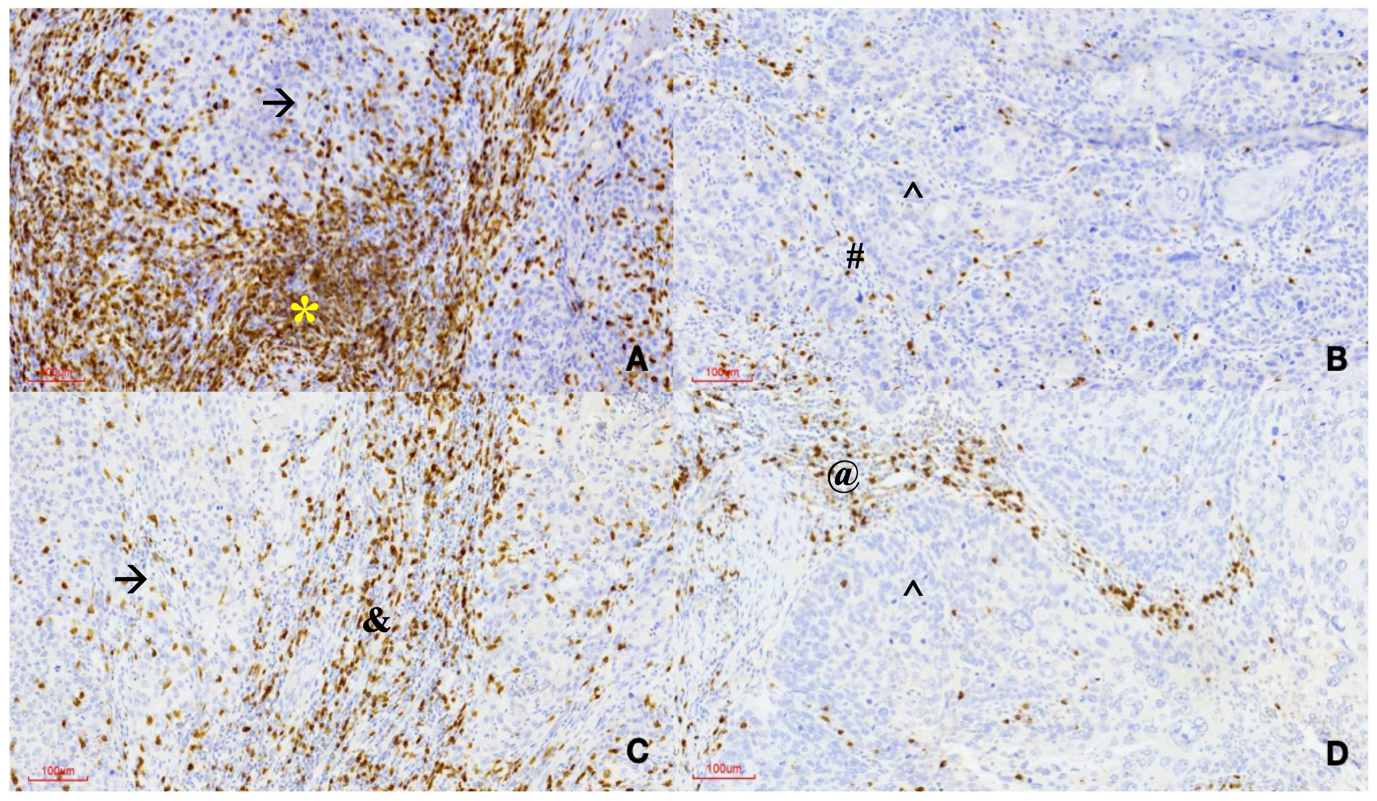


**Supplementary Figure 3**. Immunohistochemically-stained PSCC using antibodies against CD3 and CD8. A dense stromal CD3^+^ (*) and CD8^+^ (&) immune cell infiltrate is seen in **A** and **C** respectively, with a corresponding high infiltrate in the intra-tumoral areas (→). In **B** and **D**, a lower stromal infiltrate of CD3^+^ (@) and CD8^+^ (#) immune cells respectively, is seen with a corresponding lower intra tumoral infiltrate (^).
